# Nonverbal synchrony and affect in dyadic interactions

**DOI:** 10.3389/fpsyg.2014.01323

**Published:** 2014-11-24

**Authors:** Wolfgang Tschacher, Georg M. Rees, Fabian Ramseyer

**Affiliations:** Abteilung für Psychotherapie, Universitätsklinik für Psychiatrie und Psychotherapie, Universität BernBern, Switzerland

**Keywords:** nonverbal synchrony, mimicry, imitation, embodiment, coordinated body-movement, motion energy analysis (MEA), body movement

## Abstract

In an experiment on dyadic social interaction, we invited participants to verbal interactions in cooperative, competitive, and ‘fun task’ conditions. We focused on the link between interactants’ affectivity and their nonverbal synchrony, and explored which further variables contributed to affectivity: interactants’ personality traits, sex, and the prescribed interaction tasks. Nonverbal synchrony was quantified by the coordination of interactants’ body movement, using an automated video-analysis algorithm (motion energy analysis). Traits were assessed with standard questionnaires of personality, attachment, interactional style, psychopathology, and interpersonal reactivity. We included 168 previously unacquainted individuals who were randomly allocated to same-sex dyads (84 females, 84 males, mean age 27.8 years). Dyads discussed four topics of general interest drawn from an urn of eight topics, and finally engaged in a fun interaction. Each interaction lasted 5 min. In between interactions, participants repeatedly assessed their affect. Using hierarchical linear modeling, we found moderate to strong effect sizes for synchrony to occur, especially in competitive and fun task conditions. Positive affect was associated positively with synchrony, negative affect was associated negatively. As for causal direction, data supported the interpretation that synchrony entailed affect rather than vice versa. The link between nonverbal synchrony and affect was strongest in female dyads. The findings extend previous reports of synchrony and mimicry associated with emotion in relationships and suggest a possible mechanism of the synchrony-affect correlation.

## INTRODUCTION

When people are affectively moved, they tend to move accordingly – the close and bidirectional link between emotion and bodily movement (Blake and Shiffrar, 2007; Hatfield et al., 2009) constitutes the core premise of the present empirical project. This assumed link is consistent with the concept *embodiment*, which has recently received support from research in psychology and cognitive science (Gallese, 2005; Tschacher and Bergomi, 2011). Embodiment denotes the theoretical perspective that mental processes must not be viewed isolated from bodily processes; the essence of cognition is recognized in sensorimotor couplings rather than in abstract information processing. A similar focus, a ‘corporeal turn,’ is currently observed in the humanities and in philosophy (Fuchs and Jaegher, 2009; Alloa et al., 2012; Fuchs and Koch, 2014). Thus, psychology is becoming increasingly sensitized to investigate the close association between mental and bodily parameters such as body motion, gesture, or facial expression.

In a dynamical systems view, *synchronization* is a pervasive concept relevant to a large number of physical (Nicolis and Prigogine, 1977), biological (Rodriguez et al., 1999; Iacoboni, 2009), and social (Salvatore and Tschacher, 2012; Schmidt et al., 2012; Chartrand and Lakin, 2013) systems. Synchronization means that previously independent variables of a system can become entrained, i.e., increasingly correlated, thereby reducing the degrees of freedom of the system. Synchronization events are typically found in complex systems undergoing transitions from disordered states to states of higher order and coherence. Non-linear systems science describes and models the laws that underlie such pattern formation and self-organization in systems across disciplines (Haken, 1977). In the present empirical study, we focused on the combination of both, embodiment and synchronization, in investigating the aspect of coordinated body-movement of individuals interacting in dyads.

This coordinated body-movement will be termed nonverbal synchrony. We were interested whether nonverbal synchrony is a manifestation of the affective states of the individuals in interaction and how synchrony and affect relate to each other, in different types of verbal interaction.

In the context of psychotherapy dyads, synchrony was previously found associated with positive affectivity reflected by better rapport and a positive quality of the therapeutic relationship (Ramseyer and Tschacher, 2011, 2014). Rapport was conceptualized according to Tickle-Degnen and Rosenthal (1990), who identified three aspects: Mutual attentiveness, positivity, and coordination. Our present operationalization of nonverbal synchrony covers their aspect of coordination, and the affective component inherent to positivity was measured by self-report questionnaire data.

In the domain of social psychology, synchrony has been studied as behavioral imitation (chameleon effect: Chartrand and Bargh, 1999), and numerous investigations were concerned with mutual adaptation during social exchange (interpersonal adaptation: Burgoon et al., 1995), and movement entrainment (Richardson et al., 2008). Again, there is a link to emotion regulation (emotional contagion: Hatfield et al., 1994). The majority of empirical studies concerned with nonverbal synchrony have been conducted in affiliative contexts, i.e., within interactional affordances that encouraged rapport between interactants (e.g., Nelson et al., 2014). To our knowledge, direct comparisons of cooperative versus competitive interaction settings and their associations to nonverbal synchrony have almost never been systematically investigated (Bernieri et al., 1996). A rare exception is Paxton and Dale (2013a), who explicitly addressed the impact of conflict on nonverbal synchrony and found that it was disrupted in comparison to cooperative interactions. This scarcity of research on nonverbal behavior in the two opposing settings of cooperation and competition is rather surprising because the relevance of these aspects for negotiation or debate has long been discussed in social psychology (e.g., Thompson, 1990; Graziano et al., 1996; De Dreu et al., 2000; Seiter et al., 2009; Dunbar and Abra, 2010). Much research on interpersonal conflict was conducted in the clinical field of marital interaction, specifically in marital conflict resolution and its association with marital satisfaction and divorce (e.g., Gottman and Notarius, 2000). In our study, we sought to directly compare the effects of cooperative and competitive settings on nonverbal synchrony in two kinds of verbal debates.

Chartrand and Lakin (2013) report that, in various settings, people synchronize and ‘mimic’ more whenever they perceive or wish a positive relationship. For example, the frequency of mimicry behaviors is predicted by attachment traits of adults (Hall et al., 2012). Vice versa, synchrony entailed liking, cooperative behavior, and further prosocial effects. Thus, synchrony was found to be both a consequence and antecedent of prosocial behavior and positive emotions.

Several explanations were proposed for this linkage between nonverbal synchrony and affect. Synchrony between interactants may support (or result from) empathic understanding (Bavelas et al., 1987; de Waal, 2007). Synchrony may also have a communicative function, creating a shared perspective of a situation (Scheflen, 1964; Wallbott, 1996). A number of studies investigating the chameleon effect have shown that imitation has beneficial effects on relationship quality and rapport (e.g., Stel and Vonk, 2010). Studies have demonstrated that synchronized motor activity increases both cooperation and affiliation (Hove and Risen, 2009; Wiltermuth and Heath, 2009). Even simple body-movements, such as walking, are more synchronized in dyads with positive relationships (Miles et al., 2010). Most of the studies referenced above explored the effects of synchrony that occurred outside of participants’ conscious awareness. Similar findings for the case of deliberate (instructed) synchronous motor activity have been reported, demonstrating increases in, e.g., self-esteem and affiliation to interaction partners (Lumsden et al., 2014).

Many areas of human social interaction generate synchronized behavior (Vallacher and Nowak, 2009): the list includes religious settings (chorusing, dancing), sporting events (‘Mexican waves’ in the stadium), and the military (marching). These behavioral examples describe ritual processes where belongingness is negotiated and positive affect is desired. Research on interactional synchrony has likewise been conducted in diverse contexts (Davis, 1982; Bernieri and Rosenthal, 1991; Burgoon et al., 1995). We derived the interactional setting for the present experimental study from previous research on nonverbal synchrony between patient and therapist: Using the above mentioned conceptualization of synchrony, we found evidence for the association between synchrony and positive aspects of the current state of a relationship (measured at the level of single sessions of psychotherapy, i.e., micro-outcome) as well as at the level of relationship development and maintenance (measured at termination of all therapy sessions, i.e., macro-outcome). We therefore sought to extend these findings from the psychotherapy setting (Ramseyer and Tschacher, 2011) to an experimental context of dyadic verbal interactions. We created an interactional setting that provided analogous instructions for both the cooperative as well as the competitive conditions. This was achieved by instructing participants to engage in verbal discussions with the aim to either convince the other fellow participant (=competition) or with the aim to defend a shared argumentational position against a third party (=cooperation). ‘Micro-outcome’ in the present, more general context was assessed by repeated ratings of participants’ affectivity directly after interactions.

The hypotheses of the present study were fourfold. As our specific methodology was not before applied outside of clinical settings, we hypothesized that nonverbal synchrony was significantly present also in dyads of unacquainted individuals who engage in prescribed conversations (hypothesis 1, one-sided). We expected that synchrony would be associated positively to positive affect and negatively to negative affect resulting from these conversations (hypothesis 2, two-sided). We wished to assess temporal sequences, a potential indicator of the direction of causality between synchrony and affect: Is nonverbal synchrony better explained by affect ratings prior to, or subsequent to, the respective interactions where synchrony occurs (hypothesis 3, two-sided)? Finally, in an exploratory approach, we wished to model the dependence of positive and negative affect on synchrony together with interaction type, sex, age, and personality traits of the interactants; additionally, possible differential effects of synchrony in combination with sex or interaction type were tested (hypothesis 4, exploratory).

## MATERIALS AND METHODS

### SETTING

The project consisted of staged dyadic interactions between previously unacquainted persons of the same sex. Each dyad had five interactions; four concerned social or political topics of common interest, which were randomly drawn from an urn (without replacement); the fifth interaction was a fun task. For the initial four interactions, eight different topics were prepared, such as ‘do tuition fees at university make sense?,’ ‘media influences on child development,’ ‘voluntary army or conscript army?’ and similar. These topics provided the basis for verbal debates between members of each dyad. Participants of a dyad were provided with one of two different and opposing written lists of specific arguments fitting these topics, which they could read in a 2-min preparation period prior to the interaction. The dyads were given instructions for the respective interaction; two instructions encouraged cooperation and two instructions encouraged competition. Cooperation instruction 1 was to develop a shared position with the strongest arguments from the lists; the other cooperation instruction (2) additionally invoked an imagined third party against which the dyad was asked to discuss the best shared argumentation strategy. The competition instruction 1 was to argue, on the basis of each participant’s list of arguments, as convincingly as possible against the position of the interaction partner; the second competition instruction was asymmetrical, where one participant received a longer list of five strong arguments, and the other a list of two weaker arguments. The fifth interaction was a ‘fun task’ with the instruction, “Please design a five-course meal composed of dishes and drinks that both you and your interaction partner dislike.” The fun task was adapted from Chovil (1991). Our motivation for including the fun task was to investigate the relationship between affect and synchrony not only in themed discussions, but also in the engaging and humorous atmosphere reliably created by it; this assumption had been supported in the pilot phase of the present experiment. The fun-task type of cooperation was also more similar to previous work employing cooperative and competitive interactions (e.g., Bernieri et al., 1994). Given that the fun task deviated from the previous four, more highly structured, topical interactions, we always included it at the end of the experiment outside of the randomization scheme. The sequence of instructions was randomized and balanced, with 50% of dyads receiving cooperation 1 – cooperation 2 – competition 1 – competition 2 – fun task, and 50% receiving competition 1 – competition 2 – cooperation 1 – cooperation 2 – fun task.

**FIGURE 1:**
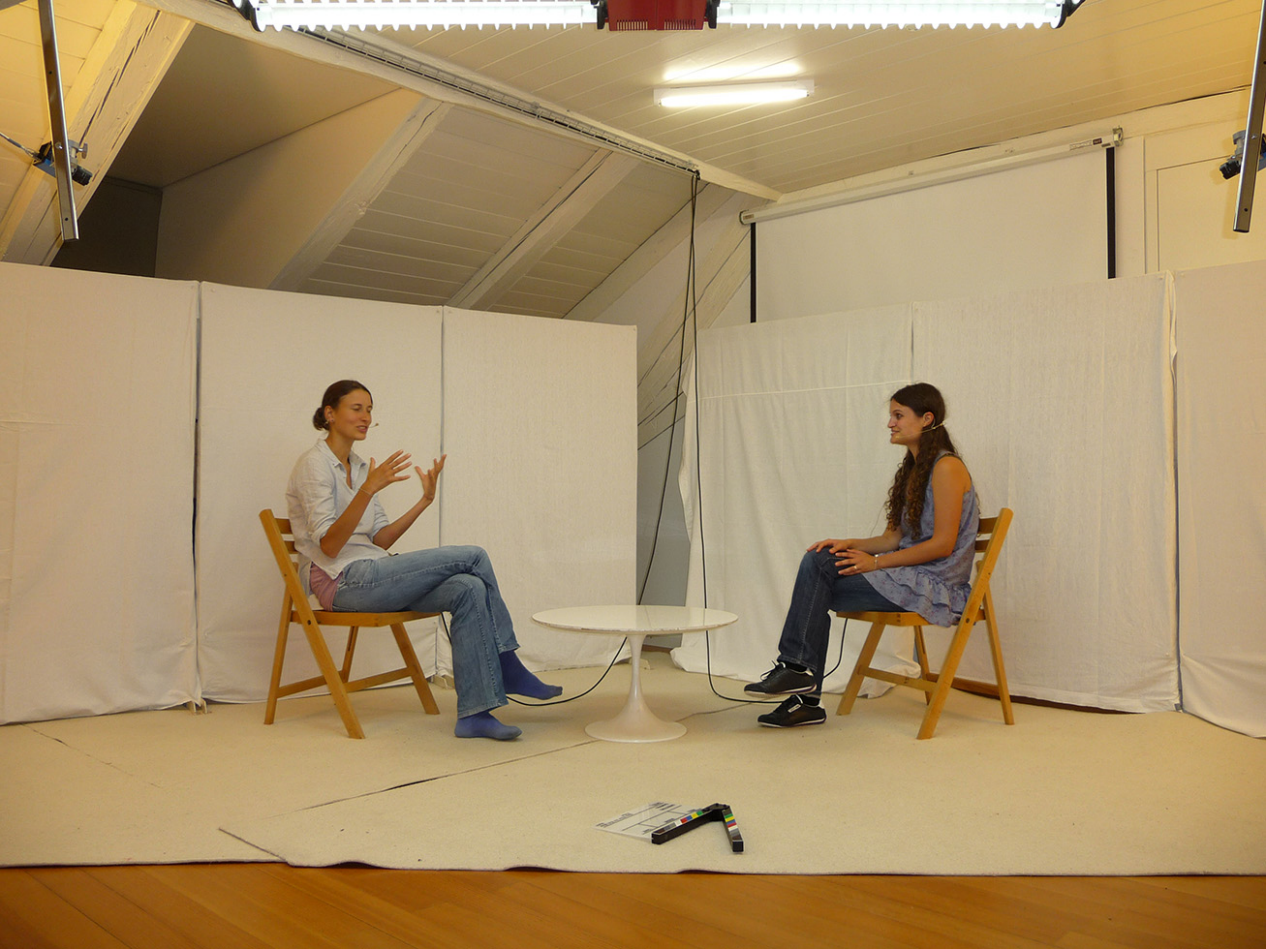
**Depiction of the experimental setup.** Top left and right, digital video cameras.

All five interactions (duration 5 min each) of a dyad were recorded using two cameras joined into a split-screen image. Prior to the experiment, it was ascertained that participants did not know each other, were fluent speakers of German, and had no current or previous psychiatric diagnosis. Participants were unaware of the specific hypotheses of the study, especially of the fact that the synchrony of movement was a variable of interest. Thus, the goals of the experiment were not disclosed in detail until the end of the experiment. Instead, participants were informed that the experiment sought to analyze processes taking place in verbal negotiations between unacquainted persons. The filming and audio recording of interactions was explained as being a prerequisite for subsequent evaluation of discussion performance. The interactions were conducted in a studio-like setting with standardized seating arrangements (**Figure 1**). The audio–visual recording of interactions was openly declared in the recruitment description and participants gave informed consent complying with Swiss ethical regulation policies. When participants arrived at the lab, a research assistant introduced them to each other and explained the sequence of events. Each person individually completed a battery of psychological measures prior to the interaction sequences.

### PARTICIPANTS

Participants (*N* = 168) were 84 women and 84 men (mean age 27.8 years, SD = 4.8), with men on average 3.37 years older [*t*(166) = 4.81; *p* < 0.0001). Participants were predominantly students or university graduates, and further persons recruited by the investigators. Fluency in German was an inclusion criterion; 87% were Swiss citizens, other participants were from Germany (9%) and other European countries; all participants were Caucasian. Participants were assigned to dyads randomly from the pool of available persons, with the rule that interactants in a dyad had the same sex and the same linguistic background, since the Swiss, German, and Austrian variants of spoken German vary considerably. Participants’ education levels were generally high, 82% had high-school levels (“Matura” degree in the Swiss schooling system), and 57% had received higher education degrees (technical or pedagogic college 12%, university graduation 45%). Participants were paid 30 Swiss francs for their time.

### MOTION ENERGY ANALYSIS (MEA) AND MEASUREMENT OF NONVERBAL SYNCHRONY

**FIGURE 2:**
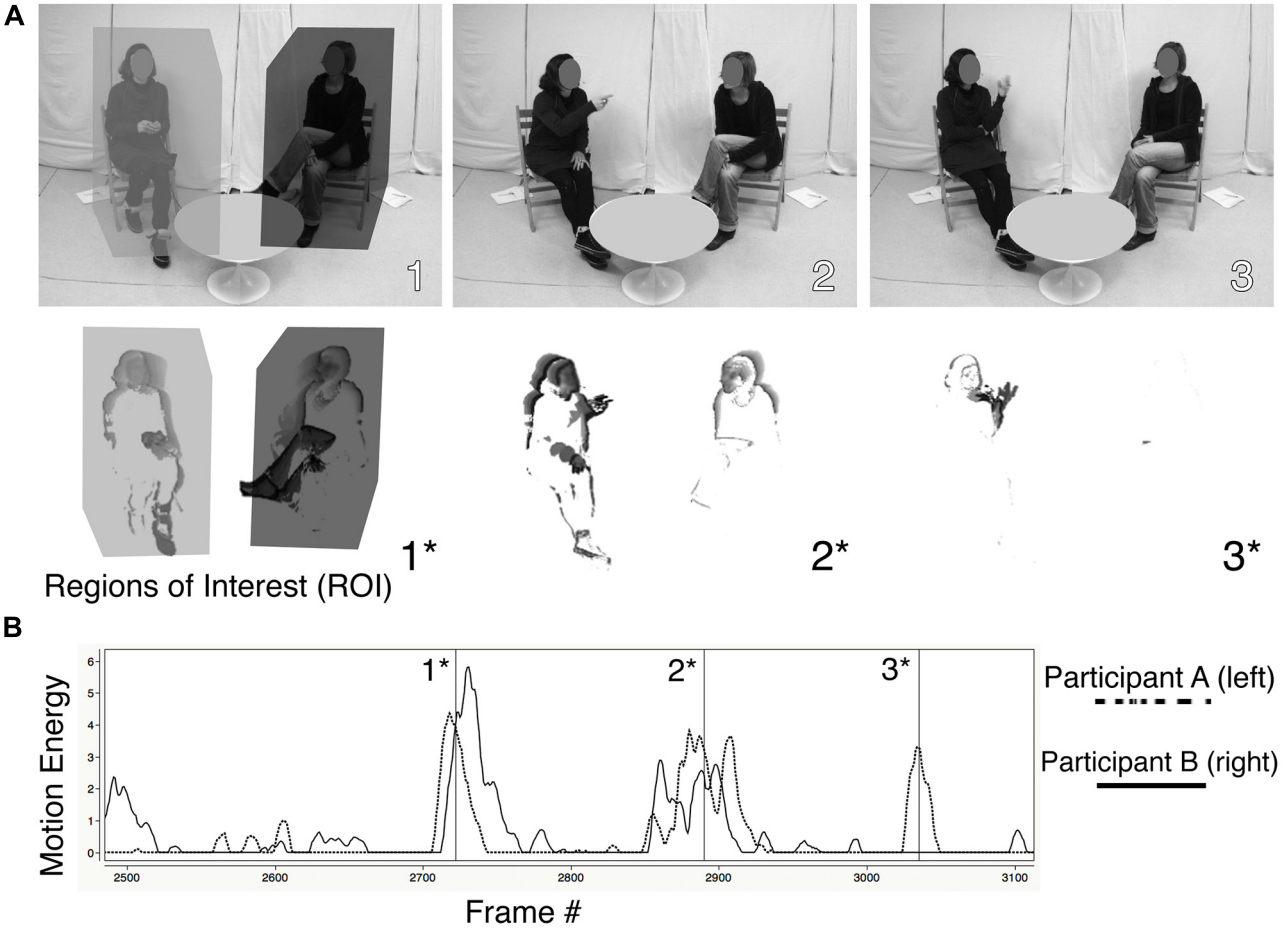
**Motion energy analysis (MEA).** (**A**; top row), consecutive frames 1, 2, and 3 of split-screen video recordings. (**A**; bottom row), corresponding images of motion energy 1*, 2*, and 3*. **(B)** time series of individual motion energies (ordinate: smoothed, *z*-standardized and threshold-adjusted motion energy values; abszissa: time in 1/10 s). The regions of interest (ROI) are shown as boxes in image 1 and 1*. In images 1* and 2*, episodes of nonverbal synchrony occur.

The idea of interactional synchrony was originally introduced by Condon and Ogston (1966), who manually coded movement changes occurring between consecutive frames of film recordings. Other methods have relied on trained judges’ evaluations (Bernieri and Rosenthal, 1991). Technical advances in digital video processing have since greatly facilitated quantification of movement based on video recordings (Grammer et al., 1997; Grammer et al., 1999; Ramseyer and Tschacher, 2006, 2011; Nagaoka and Komori, 2008; Kupper et al., 2010; Altmann, 2011; Paxton and Dale, 2013a,b). Motion energy analysis (MEA), an objective method to determine changes in movement, relies on the same principle of frame-by-frame change introduced almost five decades ago. Yet, MEA provides a cost- and time-efficient alternative to manual observer ratings because it is automated to continuously monitor the amount of change occurring in pre-defined regions of interest (**Figure 2**). The technical prerequisites for the recordings are a static camera position and stable light conditions with constant shutter-speed and aperture. Regions of interest should not overlap, and people should not occlude one another.

Motion energy analysis involves several processing steps: Digitized sequences (10 frames/s) of dyadic interactions were analyzed with commercial video-analysis software (‘softVNS’ Rokeby, 2006; ‘MaxMsp’ cycling ’74, 2006) that was customized for MEA (Ramseyer, 2008; see www.psync.ch for details). Motion energy was defined as differences in gray-scale pixels between consecutive video-frames (Grammer et al., 1999), with differences indicating body motion of the respective participant. One region of interest (ROI) was adapted to each participant, covering the entire body including the head and legs (see **Figure 2A**). Time-series of these raw pixel-changes in each ROI were then smoothed with a moving average of 0.5 s, which reduces fluctuations due to signal-distortion present in most videos. In order to account for different size regions of interest, data were z-transformed and a threshold for minimal movement was individually calculated for each interaction and each participant. Data filtered and corrected in this manner were submitted for quantification of nonverbal synchrony (see below). The objectivity of this kind of automatic movement analysis is high, i.e., MEA is observer-independent once the procedure is established. MEA provides objective and unobtrusive quantitative measures of movement dynamics.

The quantification of nonverbal synchrony was based on cross-correlations of participants’ movement time-series. In every 5-min interaction, motion energies of both participants were cross-correlated (Boker et al., 2002) in window segments of 30 s duration. In contrast to previous work with MEA in psychotherapy dyads (Ramseyer and Tschacher, 2011), the window size of 30 s was chosen to account for the relatively more dynamic and shorter turn-taking latencies in argumentative interaction compared to a psychotherapy setting. Segmentation into windows was chosen in order to take into consideration the non-stationary nature of movement behaviors. Cross-correlations for positive and negative time-lags up to 5 s in steps of 0.1 s were computed by step-wise shifting one time-series in relation to the other (50 steps in each direction, i.e., positive and negative lags). Cross-correlations were then standardized (Fisher’s Z) and their absolute values were aggregated over the entire 5-min interval of an interaction, yielding one global value of nonverbal synchrony for each of the five interactions of each dyad. The use of absolute values means that both positive and negative cross-correlations contributed positively to the 5-min synchrony measure. This strategy yields synchrony values representative of the movement coordination of an interacting dyad. These values were used as the synchrony variable in testing hypotheses 2, 3, and 4.

In order to evaluate the significance of synchrony values (hypothesis 1), a control for coincidental synchrony is needed. Bernieri et al. (1988) worked with so-called pseudointeractions “... by isolating the video image of each interactant and then pairing them with the video images of other interactants recorded in other interactions” (p. 245). Bernieri et al. (1988) were able to show significantly higher synchrony in genuine mother–child interactions than in pseudointeractions. We implemented a similar technique and generated pseudointeractions using automated surrogate algorithms (Ramseyer and Tschacher, 2010). Surrogate datasets (*n*= 100 out of each genuine dataset) were produced by segment-wise (30 s segments) shuﬄing of the original data of interactants X and Y of a dyad, then aligning movement segments of X with movement segments of Y that never actually occurred at the same time. This procedure kept the window-wise, individual progressive time structure of the real data intact but permuted the temporal location of window segments. Synchrony in pseudointeractions (i.e., pseudosynchrony) was finally calculated identically to the synchrony of the original data as described above. For the statistical comparison of synchrony versus pseudosynchrony, the synchrony values of all 100 surrogate datasets of a dyad were computed in each of the five interaction types, yielding a distribution of pseudosynchronies. Each dyad’s five genuine synchrony values were expressed as z values on the basis of the respective distribution of pseudosynchronies.

### PROCESS MEASURE OF AFFECT

Prior to and subsequent to each of the five interaction tasks, all participants rated their own momentary affective state using the *Positive and Negative Affect Scale*, PANAS (Krohne et al., 1996). The PANAS is a short standard instrument for the self-assessment of emotional states. It consists of twenty emotion adjectives (e.g., ‘active,’ ‘interested,’ ‘upset,’ ‘afraid’) that are rated on five-point scales ranging from ‘very slightly or not at all’ to ‘extremely.’ The 20 items load on two factors, positive affect and negative affect. Internal consistencies of the factors are usually found to be high, exceeding 0.80. The PANAS is a change-sensitive instrument and thus has low test-retest reliability. The positive and negative affect factors were used in hypotheses 2, 3, and 4.

### MEASURES OF PERSONALITY TRAITS

All participants filled out questionnaires covering different areas such as interactional style, interpersonal problems, personality traits, and psychological symptoms, to be used as predictors in hypothesis 4. All instruments were based on participants’ self-reports. These questionnaires were given at baseline, prior to the interactions.

The *Five Factor Personality Inventory* (NEO-FFI, Borkenau and Ostendorf, 1991; Costa and McCrae, 1992) is a multidimensional questionnaire for the self-assessment of the fundamental dimensions of personality (the postulated “Big Five” are neuroticism, extraversion, openness for new experience, agreeableness, conscientiousness). The five dimensions are measured on the basis of 60 items with five-point scales. Cronbach’s alpha of the dimensions in the German version ranged between 0.71 (openness) and 0.85, and test–retest reliabilities are commonly reported as adequate.

The *Inventory of Interpersonal Problems* (IIP, Horowitz et al., 1988, 1994) is a measure of current difficulties in interpersonal functioning. Apart from a total score indicative of the overall level of interpersonal problems, eight subscales pertaining to the circumplex model of interpersonal behavior are assessed using a five-point Likert scale (64 items ranging from 0 to 4). Horowitz et al. (1994) reported an internal consistency ranging from 0.82 to 0.94 with a 10-week test–retest reliability of 0.80 to 0.90.

The patients’ adult attachment style was measured with the *Measure of Attachment Qualities* (MAQ, Carver, 1997). The 14 items are scored on a four-point Likert scale ranging from 1 to 4. The measure provides four scales of attachment types: security, avoidance, ambivalence-worry, and ambivalence-merger. Carver (1997) reported internal consistencies of 0.69 to 0.76 and a test–retest reliability of 0.61 to 0.80 over a 6-week period.

The SCL-K-9 is a short version of the *Symptom Checklist* SCL-90-R, composed of the nine items correlating highest with the global severity index of the SCL (Franke, 1994; Klaghofer and Brähler, 2001), a measure used to assess general symptom distress, such as worries, emotional instability, being nervous. Using a five-point Likert scale, participants indicate to what extent they experienced each of nine distress symptoms in the past week. We used the mean of the items as an assessment of overall current symptomatology. For the original scales, internal consistencies between 0.63 and 0.85 with a test–retest reliability between 0.73 and 0.92 over a 1-week period were reported.

The Saarbrücker Persönlichkeitsfragebogen SPF (Saarbrücken personality questionnaire) is a reworked German version of the *Interpersonal Reactivity Index* (IRI: Davis, 1983), a questionnaire for the measurement of empathy. The SPF has four scales, perspective taking, fantasy, empathic concern, and personal distress. Paulus (2009) reported internal consistencies of 0.66 to 0.71 and good test–retest reliability.

In the present study, factor analysis was used to construct factors that represent these personality-based and clinical trait measures. The intention was to define strictly orthogonal factors that were suited as predictors in later regression analyses (hypothesis 4), thereby avoiding collinearity of predictors. The small number of factors also reduces the problem of alpha inflation of statistical modeling, since the complete set of trait data comprised 22 subscales of the available instruments (five subscales of the NEO-FFI, eight subscales of the IIP, four of the MAQ, one SCL-K-9 scale, four subscales of the SPF). Factor analysis with Maximum likelihood estimation (JMP Pro 10 statistical software) yielded six factors with an Eigenvalue > 1. Using Varimax rotation, six orthogonal factors were obtained by the linear composites of the subscales. These factors explained 57.4% of total variance. Factor loadings are given in **Table 1**. The factors (with explained variances) can be described as follows:

**Table 1:** Factor loadings after factor analysis with Varimax rotation of 22 questionnaire scales.

Scale	Factor 1 selfish-domineering	Factor 2 non-assertive-accommodating	Factor 3 cold-avoidant	Factor 4 ambivalent-troubled	Factor 5 introverted-distressed	Factor 6 open-empathic
NEO neuroticism	0.32	0.15	0.04	**0.52**	**0.56**	0.12
NEO extraversion	-0.01	0.02	**-0.49**	-0.07	**-0.52**	0.15
NEO openness	-0.06	-0.07	0.00	-0.19	-0.05	**0.61**
NEO agreeableness	**-0.60**	0.25	-0.33	-0.09	0.03	0.10
NEO conscientiousness	-0.36	-0.07	0.02	0.03	-0.11	-0.05
MAQ security	-0.04	0.05	**-0.49**	0.11	0.05	0.11
MAQ avoidance	0.10	0.13	**0.69**	0.06	0.09	-0.03
MAQ ambivalence-worry	0.01	0.08	0.08	**0.77**	0.10	-0.01
MAQ ambivalence-merger	0.09	0.11	0.04	**0.50**	0.09	-0.08
IIP domineering/controlling	**0.77**	0.13	0.21	0.16	0.03	0.03
IIP vindictive/self-centered	**0.73**	0.04	0.31	0.16	0.20	-0.12
IIP cold/distant	0.34	0.19	**0.71**	0.20	0.12	0.01
IIP socially inhibited	0.17	0.30	**0.60**	0.23	**0.47**	0.02
IIP non-assertive	0.02	**0.55**	0.32	0.11	**0.59**	0.12
IIP overly accommodating	0.00	**0.88**	0.12	0.08	0.21	0.01
IIP self-sacrificing	0.19	**0.77**	0.08	0.28	0.05	0.11
IIP intrusive/needy	**0.65**	**0.45**	-0.01	0.32	-0.06	0.16
SPF perspective taking	**-0.43**	0.13	0.11	0.00	-0.17	**0.50**
SPF fantasy	0.16	0.03	-0.13	0.05	0.20	**0.64**
SPF empathic concern	0.04	0.23	-0.21	0.21	0.06	**0.56**
SPF personal distress	0.25	0.19	0.00	0.33	**0.58**	0.12
SCL-9 global	0.31	0.26	-0.05	**0.48**	0.33	0.24

*Factor loadings exceeding 0.40 are in boldface. NEO, Five Factor Personality Inventory; MAQ, Measure of Attachment Qualities; IIP, Inventory of Interpersonal Problems; SPF, Saarbrücker Persönlichkeitsfragebogen; SCL-9, Symptom Checklist short version*.

Factor 1 selfish-domineering (12.4%): This factor describes domineering, vindictive, and intrusive IIP styles with negative NEO agreeableness and SPF perspective taking.

Factor 2 non-assertive-accommodating (10.6%): High loadings on several IIP scales, especially on overly accommodating, self-sacrificing, non-assertive, and intrusive/needy.

Factor 3 cold-avoidant (10.4%): This factor loads on IIP scales cold/distant and socially inhibited, with MAQ avoidant and insecure attachment. Lower loadings are on NEO extraversion (negative).

Factor 4 ambivalent-troubled (8.7%): This factor loads highest on MAQ ambivalent-worried and ambivalent-merging attachment, with loadings on NEO neuroticism and SCL-K-9 symptoms of psychopathology.

Factor 5 introverted-distressed (8.3%): This factor loads on IIP non-assertive and socially inhibited, SPF personal distress, NEO neuroticism and, negatively, NEO extraversion.

Factor 6 open-empathic (7.1%): This factor loads on SPF fantasy, NEO openness, and SPF empathic concern and perspective taking.

### HIERARCHICAL LINEAR MODELING

For the sake of testing hypothesis 3, we construed a two-level model with interactions (Level 1) nested within dyads (Level 2). The dependent variable was nonverbal synchrony of dyads, and the two predictors of synchrony were PANAS-defined affect of interactants in a dyad, either measured before the interaction or after the interaction. The direction of causality was estimated on the basis of temporal sequence, using the significance of these predictors. Thus, we were testing competing models of temporal relationships between synchrony and affect: If affect prior to the interaction was significant whereas affect after the interaction was not, this would speak for Granger-causality ‘affect causing synchrony.’ If affect prior to the interaction was insignificant whereas affect after the interaction was significant, this would speak for Granger-causality ‘synchrony causing affect.’

For hypotheses 2 and 4, we modeled interactions (Level 1) as nested within participants (Level 2) and nested within dyads (Level 3). The dependent variables were participants’ positive (negative) affect as measured by the PANAS. *n* = 840 measurements were planned (168 participants × 5 interactions), with three PANAS measurements missing due to data loss (hence, *n* = 837 in **Tables 2** and **3**). For the exploratory assessments of hypothesis 4 we tested different explanatory variables as predictors of affect in a systematic modeling procedure, separately for participants’ positive and negative affect. We used a step-up procedure, exploring different combinations of predictors (i.e., models). We evaluated all models in terms of Akaike’s information criterion (AIC) to select the best model; we used the software package JMP Pro 10 (SAS Institute Inc, Cary, NC, USA) for computation of the corrected AIC (AICc) and for all further statistical analyses. We applied mixed-effects analysis to explain the variance of the dependent variable ‘positive (negative) affect’ by the following fixed effects (i.e., predictors): ‘Synchrony,’ ‘Interaction type,’ ‘Interaction type × Synchrony,’ ‘Age,’ ‘Sex,’ ‘Sex × Synchrony,’ ‘Factor 1, 2,..., 6.’ In all models, ‘Participant’ and ‘Dyad’ were entered as random effects, which defined the dependency structure inherent to this hierarchical dataset (Raudenbush and Bryk, 2002). The best-fitting and most parsimonious model was selected with the following procedure: We incrementally entered the predictors (fixed effects) in the sequence of the list above. Statistical significance (*p* < 0.05) of the entered predictor was applied as a criterion to either keep the current predictor and add the following predictor, or skip the current predictor and enter the following predictor. In this manner, nine models were computed for the dependent variable ‘Positive affect’ and the same number for ‘Negative affect.’ Finally, AIC was used, a common approximation to model evidence. The AIC includes both an accuracy and complexity term, in other words, it identifies the most accurate model that can also provide a parsimonious explanation for observed data. Smaller AIC indicates the better model. The respective AIC-optimal models are printed bold in the resulting tables below.

**Table 2:** Mixed effects models of *N*168 participants interacting in 84 dyads. Dependent variable, PANAS positive affect.

	Model 1 (*n* = 837)	Model 2 (*n* = 837)	Model 3 (*n* = 837)	Model 4 (*n* = 837	Model 5 (*n* = 837)	Model 6 (*n* = 837)	Model 7 (*n* = 837)	Model 8 (*n* = 837)	Model 9 (*n* = 837)
**Fixed Effects**
Synchrony		*t* = 3.65***	*t* = 3.29**	*t* = 3.52***	*t* = 3.51***	*t* = 3.47***	*t* = 3.73***	*t* = 3.75***	*t* = 3.71***
Interaction type			*F* = 10.3****	*F* = 13.3****	*F* = 13.3****	*F* = 13.3****	*F* = 14.1****	*F* = 14.1****	*F* = 14.1****
Interaction type × Synchrony				*F* = 4.86**	*F* = 4.86**	*F* = 4.88**	*F* = 4.41*	*F* = 4.48*	*F* = 4.47*
Age					*t* = 0.18				
Sex[male]						*t* = 1.72	*t* = 1.72	*t* = 1.89	*t* = 1.28
Sex[male] × Synchrony							*t* = -3.62***	*t* = -3.56***	*t* = -3.60***
Factor 1 selfish-domineering								*t* = .71	
Factor 2 non-assertive-accommodating								*t* = -.99	
Factor 3 cold-avoidant								*t* = -1.63	
Factor 4 ambivalent-troubled								*t* = -.95	
Factor 5 introverted-distressed								*t* = -2.42*	*t* = -2.84**
Factor 6 open-empathic								*t* = 1.57	
**Random Effects**
Participant [Dyad] (% variance)	48.85	49.41	50.04	50.58	50.89	51.11	51.63	51.63	51.68
Dyad (% variance)	16.27	15.95	16.06	15.54	15.33	14.65	14.67	14.47	13.28
Whole model variance (%)	71.23	71.76	72.64	72.98	72.99	72.98	73.49	73.49	73.47
AIC	1385.4	1373.6	1369.1	1361.0	1370.3	1364.2	1352.6	1374.0	**1350.6**

Factor, orthogonal factors of participants’ traits; AIC, Akaike’s Information Criterion. PANAS, Positive and Negative Affect Scale. AIC minimum printed in boldface. For each model, fixed effects estimates, random effects estimates, whole model variance and AIC are listed (top to bottom). *p< 0.05; **p< 0.01; ***p< 0.001; ****p< 0.0001.

**Table 3:** Mixed effects models of *N* = 168 participants interacting in 84 dyads. Dependent variable, PANAS negative affect.

	Model 1 (*n* = 837)	Model 2 (*n* = 837)	Model 3 (*n* = 837)	Model 4 (*n* = 837)	Model 5 (*n* = 837)	Model 6 (*n* = 837)	Model 7 (*n* = 837)	Model 8 (*n* = 837)	Model 9 (*n* = 837)
**Fixed Effects**
Synchrony		*t* = -2.28*	*t* = -0.62	*t* = -0.60	*t* = -0.61	*t* = -0.62	*t* = -0.57		
Interaction type			*F* = 50.9****	*F* = 46.6****	*F* = 50.9****	*F* = 50.9****	*F* = 51.1****	*F* = 53.73****	*F* = 53.70****
Interaction type × Synchrony				*F* = 0.46					
Age					*t* = 0.66				
Sex[male]						*t* = 0.17	*t* = 0.16		
Sex[male] × Synchrony							*t* = -0.65		
Factor 1 selfish-domineering								*t* = 2.56*	*t* = 2.61*
Factor 2 non-assertive-accommodating								*t* = 3.79***	*t* = 3.96***
Factor 3 cold-avoidant								*t* = 0.00	
Factor 4 ambivalent-troubled								*t* = 4.13****	*t* = 4.37****
Factor 5 introverted-distressed								*t* = 1.14	
Factor 6 open-empathic								*t* = 1.58	
**Random Effects**
Participant [Dyad] (% variance)	39.99	40.28	44.28	44.22	44.61	44.12	44.06	33.00	31.83
Dyad (% variance)	3.13	2.79	3.26	3.30	2.98	3.61	3.73	7.92	9.40
Whole model (% variance)	52.03	52.32	58.95	59.00	58.97	58.97	59.02	58.57	58.53
AIC	385.9	383.1	306.3	309.3	316.9	314.3	316.5	308.5	**288.3**

Factor, orthogonal factors of participants’ traits; AIC, Akaike’s Information Criterion; PANAS, Positive and Negative Affect Scale. AIC minimum printed in boldface. For each model, fixed effects estimates, random effects estimates, whole model variance and AIC are listed (top to bottom). *p< 0.05; **p< 0.01; ***p< 0.001; ****p< 0.0001.

## RESULTS

Nonverbal synchrony did not vary significantly across the four debate conditions that realized cooperation or competition [*F*(1,83) = 0.082; *p* = 0.775], but it significantly increased across all five interactions [it was higher in the final interaction, the fun task; *F*(1,83) = 17.993; *p* < 0.0001]. Within the 5-minute tasks, there was no effect of time on synchrony across all 30-second windows [*F*(9,3986) = 0.933; *p* = 0.495].

### HYPOTHESIS 1

The comparison of synchrony with pseudosynchronies derived from shuﬄed data demonstrated that nonverbal synchrony was significantly present at an above-chance level in this sample of 84 dyads and in all of the five interaction conditions. The mean *z* values for genuine synchrony were 0.41 (cooperation 1), 0.71 (cooperation 2), 0.74 (competition 1), 0.78 (competition 2), and 1.11 (fun task). These mean *z* values are identical to effect sizes (Cohen’s *d*), i.e., they demonstrate moderate to strong effects. When the five interaction conditions are collapsed in three conditions, mean *z* values were 0.56 (cooperation), 0.76 (competition), and 1.11 (fun task). The fun task showed a significantly higher synchrony than both competition (*p* < 0.05; *d* = 0.30 for the difference) and cooperation (*p* < 0.001; *d* = 0.49).

The mean genuine synchrony values were 0.168 (cooperation 1), 0.170 (cooperation 2), 0.174 (competition 1), 0.178 (competition 2), and 0.195 (fun task). In three categories, genuine synchrony ranged from 0.169 (cooperation), to 0.176 (competition), and 0.195 (fun task); the three categories of genuine synchrony were all significantly different from each other (*p* < 0.0001; *d* = 0.63 and 0.86). There was no general increase or decrease of synchrony over time in the initial four interactions whose sequence was randomized.

### HYPOTHESIS 2

Hierarchical linear models were computed for positive affect (**Table 2**) and negative affect (**Table 3**). Individual affect ratings were used. In both tables, model 2 refers to the modulation of affect by the dyad’s synchrony alone. As expected, it was found that nonverbal synchrony positively predicted positive affect (*t* = 3.65, *p* < 0.001) and predicted reduced negative affect (*t* = -2.28, *p* < 0.05).

### HYPOTHESIS 3

Hierarchical linear models were computed for nonverbal synchrony (dependent variable) explained by positive affect prior to the respective interaction and by positive affect after this interaction; affect ratings were the averages of the two dyad members in the interaction (**Table 4**, Model a). The procedure was repeated with negative affect prior to, and after, an interaction (**Table 4**, Model b). We found that positive affect after the interactions was a significant effect in the model (*t* = 2.52, *p* < 0.05) whereas positive affect prior to the interactions was not (*t* = -0.16, *p* = 0.87). Negative affect after the interactions was likewise significant in Model b (*t* = -1.98, *p* < 0.05) whereas negative affect prior to the interactions was not predictive (*t* = 0.08, *p* = 0.93). This supported the assumption that, in the present sample, positive or negative affect may have been caused by synchrony rather than that affect caused synchrony.

**Table 4:** Mixed effects models of *N* = 84 dyads in five interaction conditions. Dependent variable, nonverbal synchrony.

	Model a (*n* = 420)	Model b (*n* = 420)
**Fixed Effects**
PANAS positive before	*t* = -0.16	
PANAS positive after	*t* = 2.52*	
PANAS negative before		*t* = 0.08
PANAS negative after		*t* = -1.98*
**Random Effect**
Dyad (% variance)	6.30	5.72
Whole model variance (%)	12.66	11.15
AIC	-1705.9	-1705.3

For both models, fixed effects estimates, the random effect estimate, whole model variance, and AIC are listed (top to bottom). AIC, Akaike’s Information Criterion; PANAS, Positive and Negative Affect Scale. *p< 0.05.

### HYPOTHESIS 4

The preferred direction of causality suggested by the test of hypothesis 3 advised to treat positive and negative affect as the dependent variables in further explorations. As in the test of hypothesis 2, individual affect ratings were used. Hierarchical linear models with all predictors entered in a sequential fashion are shown in **Tables 2** and **3** (to simplify the tables, *F* values are given for the categorical predictor ‘Interaction type,’ for the specific *t* values see the following text).

Participants’ positive affect was best explained by Model 9 of **Table 2**, by the predictors ‘Synchrony,’ ‘Interaction type’ and, negatively, the personality factor 5 ‘introverted-distressed.’ As for ‘Interaction type,’ the competition condition produced significantly higher positive affect (*t* = 4.97, *p* < 0.0001), and the cooperation condition significantly lower positive affect (*t* = -2.79, *p* < 0.01), both relative to the fun task. Additionally, the interactions ‘Interaction type × Synchrony’ and ‘Sex × Synchrony’ were significant predictors: The single tests of the former interaction showed that in the cooperation condition, synchrony was linked less with positive affect as compared to the fun task condition (*t* = -2.94, *p* < 0.01), whereas competition and fun task resulted in similar linkages of synchrony and positive affect (*t* = 0.61, *p* = 0.55). The latter interaction ‘Sex × Synchrony’ means that in male dyads, synchrony was less closely linked with positive affect than in female dyads (*t* = -3.60, *p* < 0.001, see **Table 2**).

Negative affect of interactants was predicted by a different combination of independent variables (Model 9 in **Table 3**): Again, interaction type was a significant predictor. Interaction type fully mediated the influence of synchrony on negative affect, since synchrony was (negatively) predictive in Model 2, but lost significance as soon as interaction type was also entered. The competition condition of ‘Interaction type’ was associated with significantly higher negative affect than the fun task (*t* = 10.05, *p* < 0.0001), the cooperation condition and the fun task were not statistically different (*t* = -0.49, *p* = 0.62). Three trait factors contributed to negative affect: Factor 1 (selfish-domineering), Factor 2 (non-assertive-accommodating) and Factor 4 (ambivalent-troubled). In addition to the predictive value of interaction type, we found a significant decrease of negative affect over the course of the experiment.

## DISCUSSION

In the present study, we explored nonverbal synchrony in same-sex dyads that engaged in discussions of prescribed topics. The method used was motion energy analysis (MEA), an objective and automatized movement analysis of video recordings. MEA has been previously applied only to clinical samples (movement deficits in schizophrenia: Kupper et al., 2010; patient–therapist synchrony in psychotherapy sessions: Ramseyer and Tschacher, 2011). Here, we implemented the same measure with a different design and context, the first prospective application of MEA in healthy adults. Based on the analogous methodological setup, we showed here that nonverbal synchrony was present to an even higher extent than in the psychotherapy sample, with most effect sizes exceeding the moderate range. Effect sizes (Cohen’s *d*) between *d* = 0.50 and 0.59 were reported in psychotherapy dyads (Ramseyer and Tschacher, 2011). The effect sizes in the present sample were between 0.56 for interactions in the cooperation condition, to 0.76 in competitive discussions, and 1.11 in a fun task. Hence it was found that, other than might have been expected (Bernieri et al., 1994; Paxton and Dale, 2013b), (mildly) competitive conversations were more synchronized than conversations with cooperation instructions. However, the comparison with previous studies assessing both cooperative and competitive interactions should be made with caution: In our experiment, the instructions for cooperative and competitive debates were of a similar nature: The activity in both conditions was identical, only the focus (“convince your partner” versus “convince a third party”) was modified by our instructions. The available eight topics in our study were used for both cooperative and competitive discussions. This kind of similarity is clearly different from Bernieri et al.’s (1994) vacation-trip planning (cooperative) versus debate (competitive) instructions, or from Paxton and Dale’s (2013b) affiliative (“find and discuss media that both enjoy”) and argumentative (“try to convince each other of your opinion”) instructions.

Competition realized in the present project was linked with the highest ratings for both positive and negative affect, thus competition was affectively arousing and not perceived entirely negative. On the other hand, cooperation realized in this project was linked with the lowest ratings for positive affect (*d* = -0.22 in comparison to competition), which could be interpreted as a sign that our implementation of a cooperative debate was less prone to elicit arousal in participants. Our fun task was more similar to the trip-planning and media-discussion activities implemented in Bernieri et al.’s (1994) and Paxton and Dale’s (2013b) experiments: In line with their findings, our fun task ranked highest in synchrony and lowest in negative affect (*d* = -0.66 in comparison to competition). These differences suggest that task affordances were a driving force for synchrony and also influenced affect in this experiment.

In general, we found objective evidence of nonverbal synchrony in dyadic interactions among healthy individuals previously unknown to each other, and were able to quantify the (considerable) magnitude of synchrony using effect sizes. Nonverbal synchrony in this project was obtained inconspicuously, i.e., participants were blind to our focus on the social synchronization of their body movement. This finding was possible on the basis of MEA methodology. It is a merit of this and similar frame-differencing approaches (e.g., Delaherche et al., 2012) that they are independent of ratings by researchers, and can be applied in settings where participants may move without restrictions. Hence, the use of computerized video analysis offers a tool to improve the reproducibility of social psychology findings (Yong, 2012; Nelson et al., 2014).

Affect was associated with nonverbal synchrony in the expected way: more synchronous interactions were entailed by higher positive affect and lower negative affect. Comparing the association between synchrony and affect directly before and affect directly after a conversation, we found that the present data favored an interpretation that synchrony entailed affect and not vice versa. This may indicate the predominant direction of causality between synchrony and affect in the present sample. Synchrony was not merely an expression of participants’ affective states. Instead, our findings are rather consistent with the interpretation that synchrony caused affect, with a moderate to large effect size. To our knowledge, this is the first project where the two directions of causality were compared within the same dataset. Albeit not resulting from an experimental design, this finding provides new information on the generative mechanisms responsible for the synchrony-affect correlation.

Nonverbal synchrony measured by MEA has the great advantage of being objective, reproducible, and independent of the (supposed) meanings of nonverbal gestures and postures – we created a content-independent measure in the spirit of non-linear systems theory. As psychologists, however, we wished to assess what the measurements ‘mean’: The exploratory analyses (hypothesis 4) corroborated the strong link between synchrony and positive affect in dyadic interactions (hypothesis 2) when, importantly, all participants are unaware of the measurement of nonverbal synchrony. This indicates that the embodiment of dyadic interactions, as represented by correlated body movement, is an important contributor to positive affect originating from these interactions, and hence to the emotional quality of the dyadic relationship. We showed that the direct link between bodily variables and positive affect was not explained by personality traits, sex, or age of the interacting individuals. The influence of synchrony was partially moderated by sex of participants and the type of the interactions, because the connection between synchrony and positive affect was especially enhanced in female dyads, as well as in competitive and humorous/affiliative conversations. Yet none of the various explored predictors and interactions of predictors reduced the direct linkage between synchrony and positive affect.

### LIMITATIONS

As described in the Section “Materials and Methods,” MEA does not assess the qualitative aspects of nonverbal behavior, which means that MEA does not take into account qualitative features such as participants’ facial expression (e.g., smiling) or posture (e.g., approaching vs. disengaging postures). Future analyses based on observer codings will shed more light on the association of these qualitative features of nonverbal behavior and their relation to synchrony and affect. Further analytic avenues could be opened up by using Actor-Partner-Interdependence Models (APIM; Kenny et al., 2006). The present study has limitations in the causality assessment (hypothesis 3) since its design rests on temporal, not experimental, inference (i.e., causality inferred from temporal sequence). We interpreted that nonverbal synchrony Granger-caused positive and inhibited negative affect, yet in principle third variables such as the motivational valence of the tasks and topics may have influenced the association between synchrony and affect. Nevertheless, Granger causality constitutes the only design that allows checking both causal directions in the same dataset, which appears preferable to conventional designs such as comparing two experimental groups. The included sample has certain additional limitations as we recruited an academically advanced sample not representative of the general population. Also, we formed only same-sex dyads owing to statistical power considerations and to previous research indicating erratic findings in opposite-sex dyads – sex complicates interaction. The restriction to same-sex dyads obviously narrows the generalizability of the findings. Finally, we did not fully randomize the temporal sequence of interaction type in the experimental design because we expected the fun task to have extremely divergent implications when positioned as first versus final task. *Post hoc* analyses, however, showed that negative affect may have significantly dwindled over time. Thus, the predictors ‘temporal sequence’ and ‘interaction type’ were not fully disentangled, and in the modeling of negative affect the temporal sequence would probably be an important fixed effect. A minor problem was the inclusion of somewhat older male than female participants, since age was explicitly controlled for and found to be an insignificant predictor in all models. The experimental context in the project implied that the monetary compensation was given irrespective of a participant’s performance in the interactions. Thus, competitive and cooperative behavior was not specifically rewarded, which probably affected the fidelity of these two interaction types, and may in part explain the unexpected synchrony we found in competitive conversations.

## CONCLUSIONS

Concerning conceptualization of the main construct of this study, ‘synchrony’ is an appropriate and neutral concept, and in our opinion preferable to concepts such as ‘mimicry’ or ‘imitation’: Like in the present study, synchrony is commonly found to occur unintentionally, without the awareness of interactants, hence quite unlike what a ‘mime’ in ‘mimicking’ or ‘imitating’ would do. As stated in the introduction, we believe that a tendency toward synchronization may be derived from general assumptions and even laws concerning the self-organization of complex systems (Tschacher and Haken, 2007; Haken and Tschacher, 2010). Synchrony emerges independent of interactants’ intentions or imitation goals. This notion is supported by the fact that synchrony in social contexts occurs spontaneously and usually unnoticed by interactants. Based on theoretical considerations of complex systems theory applied to psychology (Kyselo and Tschacher, 2014), we would theorize that it is the individually varying affordance, or motivational valence, of the respective discussion topics that may account for the not further explicated variance components of the random effect ‘participant’ (cf. **Tables 2** and **3**). Future research should therefore increasingly incorporate qualitative and even narrative analyses of nonverbal behavior and its associations with the context and content of conversations.

In both practical and scientific applications, we believe that MEA methodology is suitable for the exploration of a wide range of social encounters where coordinative processes such as nonverbal synchrony occur. It offers objective measures for analyzing individual movement as well as for assessing synchrony in dyads or more complex social aggregates. Given today’s high availability of video recordings, MEA greatly facilitates research into nonverbally embodied aspects of emotion and communication.

## Conflict of Interest Statement

The authors declare that the research was conducted in the absence of any commercial or financial relationships that could be construed as a potential conflict of interest.
